# Filling the Disaster Data Gap: Lessons from Cataloging Singapore’s Past Disasters

**DOI:** 10.1007/s13753-021-00331-z

**Published:** 2021-02-03

**Authors:** Yolanda C. Lin, Feroz Khan, Susanna F. Jenkins, David Lallemant

**Affiliations:** 1 Department of Geography and Environmental Studies, University of New Mexico, Albuquerque, NM 87131 USA; 2 Asian School of the Environment, Nanyang Technological University, Singapore, 639798 Singapore; 3 Earth Observatory of Singapore, Singapore, 639798 Singapore; 4 Disaster Resilience Leadership Academy, Tulane University, New Orleans, LA 70112 USA

**Keywords:** Catalog, Database, Data gap, Disasters, Resilience, Singapore

## Abstract

International disaster databases and catalogs provide a baseline for researchers, governments, communities, and organizations to understand the risk of a particular place, analyze broader trends in disaster risk, and justify investments in mitigation. Perhaps because Singapore is routinely identified as one of the safest countries in the world, Singapore’s past disasters have not been studied extensively with few events captured in major global databases such as EM-DAT. In this article, we fill the disaster data gap for postwar Singapore (1950–2020) using specified metrics through an archival search, review of literature, and analysis of secondary sources. We present four key lessons from cataloging these events. First, we expand Singapore’s disaster catalog to 39 events in this time period and quantify the extent of this data gap. Second, we identify the mitigating actions that have followed past events that contribute to Singapore’s present-day safety. Third, we discuss how these past events uncover continuities among vulnerability bearers in Singapore. Last, we identify limitations of a disaster catalog when considering future risks. In expanding the disaster catalog, this case study of Singapore supports the need for comprehensive understanding of past disasters in order to examine current and future disaster resilience.

## Introduction

Most countries in Southeast Asia face risks from natural hazards of many kinds, including earthquakes, volcanoes, typhoons, landslides, droughts, and flooding. Singapore, however, is almost uniquely sheltered from many of these potential disasters, and in comparison to most of its neighboring Southeast Asian countries it has a strikingly small record of disasters. According to the 2018 INFORM Global Risk Index, Singapore ranks 190 out of 191 when assessed for exposure to natural hazards, all of which is accounted for by flooding and earthquake hazard (INFORM 2018). Similarly, Singapore falls at the very bottom of the list—191 out of 191 countries and regions—when evaluated for overall risk of humanitarian crisis and disasters. Singapore also routinely tops the lists of safest places to live (Mousavizadeh et al. 2016; The Economist Intelligence Unit 2017).

In international disaster databases, the underlying sources of much of these analyses, we find that past Singapore disasters are under-recorded. For example, in EM-DAT, an international disaster database established in 1988 and maintained by the Centre for Research on the Epidemiology of Disasters covering 1900 to the present day, there are just nine records for Singapore, with the first record appearing for 1986 (CRED n.d.). Other databases and compiled sources offer similarly low numbers (for example, GLIDEnumber 2.0 (GLIDEnumber n.d.), a database managed by the Asian Disaster Reduction Centre (ADRC), with only five records for Singapore), or exclude Singapore entirely [for example, DesInventar Sendai (UNDRR 2015a)]. Thus, Singapore’s past disasters have not been studied extensively, though these past disasters have distinctly shaped the country’s current risks through policy and other interventions.

While the under-representation of past disasters and the issue of an incomplete record is not unique to Singapore (Jennings 2011; Osuteye et al. 2017), focusing on disasters in Singapore provides a valuable opportunity to address this gap for a country that has historically been understood to be safe and nearly free from disaster. Disaster data gaps like these create significant challenges for risk reduction practice and comparative studies, but a multidisciplinary approach to identifying and cataloguing these events can address some of these challenges and make global disaster databases more accurate and relevant (Osuteye et al. 2017). For example, a fuller catalog of disasters in Singapore can provide a validation point for more regional analysis methods, while the application of such a cataloguing process elsewhere could highlight more past disasters to improve global methods such as trend analysis. This work provides a key data point and a methodology to correct for omissions in existing disaster databases and validate estimates of missing records.

A more complete catalog of past disasters is also fundamental to meeting the four priorities of the Sendai Framework for Disaster Risk Reduction 2015–2030 (SFDRR) of understanding disaster risk, managing disaster risk, investing in disaster reduction, and enhancing disaster preparedness (UNDRR 2015b). More fundamentally, addressing the “disaster data gap” supports the calls made in the SFDRR for increasing the availability of risk information and assessments “for the people,” among other priorities (UNDRR 2015b; Maini et al. 2017; Bennett 2020; Kelman 2020; Wisner 2020). The data gap has several important dimensions: the lack of inputs in data-poor contexts (Leidig and Teeuw 2015; Osuteye et al. 2017), the incompleteness of international catalogs and datasets (Jennings 2011), and the inherent challenges with measuring disaster impacts such as mortality or health impacts (Green et al. 2019). Our work focuses on improving the completeness of a particular catalog through a targeted search of relevant disaster data sources.

This study sought to fill a gap in the literature on past Singaporean disasters and describe the lessons learned through expanding Singapore’s disaster catalog. Building a case study for Singapore supports the need for comprehensive understanding of past events in order to examine current and future disaster resilience and preparedness in Singapore. Such comprehensive analysis also enables and justifies investments in mitigation for both Singapore and other contexts looking to learn from Singapore’s experience. This will be of interest to a wide range of stakeholders, including the disaster risk research community, insurance, governments, and communities and their organizations.

We first present the methods used to compile the past disasters of post-World War II Singapore to the present day (Sect. 2). This is followed by a summary of the past disasters in Sect. 3, grouped by disaster type. In Sect. 4, we discuss the four lessons that emerged from building this catalog: (1) the quantitative extent of the data gap on Singapore disasters; (2) past disasters as drivers of policy and mitigating action, which contribute to present-day safety; (3) existing vulnerabilities magnified by disaster events; and (4) limitations of a disaster catalog. We conclude in Sect. 5 with recommendations for applications of this disaster catalog and the merits of an extensive search in other places.

## Method

We include a detailed review of the past disasters of modern, postwar Singapore (1950–2020). This disaster catalog includes any event that meets at least one of the following conditions: (1) is recorded in at least one public disaster database; (2) meets at least one of the four quantitative EM-DAT disaster definitions; and/or (3) has been included in former collections of past Singapore disasters. The metrics are encoded in Table 1.

**Table 1:** At least one of the metrics included in this table must be met for inclusion in this past disaster catalog

Metric code	Metric description
1a	Inclusion in existing public disaster database: EM-DAT
1b	Inclusion in existing public disaster database: GLIDEnumber2.0
2a	10 or more people reported killed
2b	100 or more people reported affected
2c	A call for international assistance
2d	Declaration of a state of emergency
3	Inclusion in previously published disaster collection

For metric 1, three public international databases were considered: (a) EM-DAT, described in the previous section; (b) GlideNumber 2.0, a database that assigns a Global Identifier Number to disasters to better consolidate disaster information worldwide (GLIDEnumber n.d.); and (c) DesInventar Sendai, a database managed by the United Nations Office for Disaster Risk Reduction (UNDRR 2015a). However, DesInventar Sendai has no available records for Singapore and thus is not included in the following analysis. The two database sources included in meeting metric code (1) are the EM-DAT database and GLIDEnumber2.0, included in Table 1 as metrics 1a and 1b respectively.

As a second qualifying metric, we apply the EM-DAT definition of disaster: “a situation or event which overwhelms local capacity, necessitating a request to the national or international level for external assistance, or is recognized as such by a multilateral agency or by at least two sources, such as national, regional, or international assistance groups and the media” (CRED n.d.). Quantitatively, their disaster criteria require that such an event has one or more of the following characteristics: (a) 10 or more people reported killed; (b) 100 or more people reported affected; and (c) a call for international assistance or (d) declaration of a state of emergency. This second metric is meant to capture the subset of events that meet the EM-DAT definition, but are not currently included as a record. This definition can be limiting and exclude events that may still be considered disastrous to individuals or communities, but nonetheless provides a useful baseline threshold.

A third qualifying metric was added to this search of past disasters to allow for the inclusion of events that have had a lasting impression of “disaster” in Singapore’s collective memory. This last requirement is qualitative, but important to capture the perception of disaster in the context of Singapore. As such, we have drawn from other compiled collections of disaster in Singapore such as published volumes by local organizations, oral historians, and national archives. We include five sources of this kind: disaster compilations published by *The Straits Times*, the primary local news outlet; a book chapter by Lai and Tan (2015) on past disasters and disaster risk management in Singapore; a book about the Singapore environment (Friess and Oliver 2015); a compilation of Singapore’s past flu pandemics (Lee et al. 2008); and archival research and oral histories on Singapore’s history of fires in urban settlements and kampongs (Loh 2009). *The Straits Times* disaster compilations (The Straits Times 1984; The Straits Times 1986) were each published after a new disaster event (following the one year anniversary of the Sentosa Cable Car accident in 1983 and the Hotel New World collapse in 1986).

In addition to the identified databases and disaster compilations, we use national newspaper and oral history archives (National Library Board Singapore 2020), including those of *The Straits Times* (Singapore’s paper of record) stored in NewspaperSG (National Library Board Singapore 2019), publicly available archives of Singapore’s Civil Defense Force, which includes major public emergencies (SCDF 2018c), and academic publications to determine whether an event has met any defined quantitative criteria from metric 2. These sources are also used to supplement information on already-identified disasters that have qualified for inclusion in this catalog by metric 3. Several events were uncovered using this search of *The Straits Times*, most notably the string of urban fires in the 1950s and 1960s.

After collecting past disaster events through these metrics, each event is categorized as one of the following: (1) flood, (2) drought, (3) haze (a regional term referring to air pollution), (4) health, and (5) civil emergency. We then provide a summary of each event. When available, we include information on fatalities, persons affected, economic impact, domestic and/or international response, and legacy on policy. These categories have been identified from the data specifically for Singapore and are not an exhaustive list of possible disaster types. In the case of a future occurrence of a different type of disaster—for example, a typhoon—an additional category would need to be introduced.

## Past Disasters of Singapore Results

**Fig. 1:**
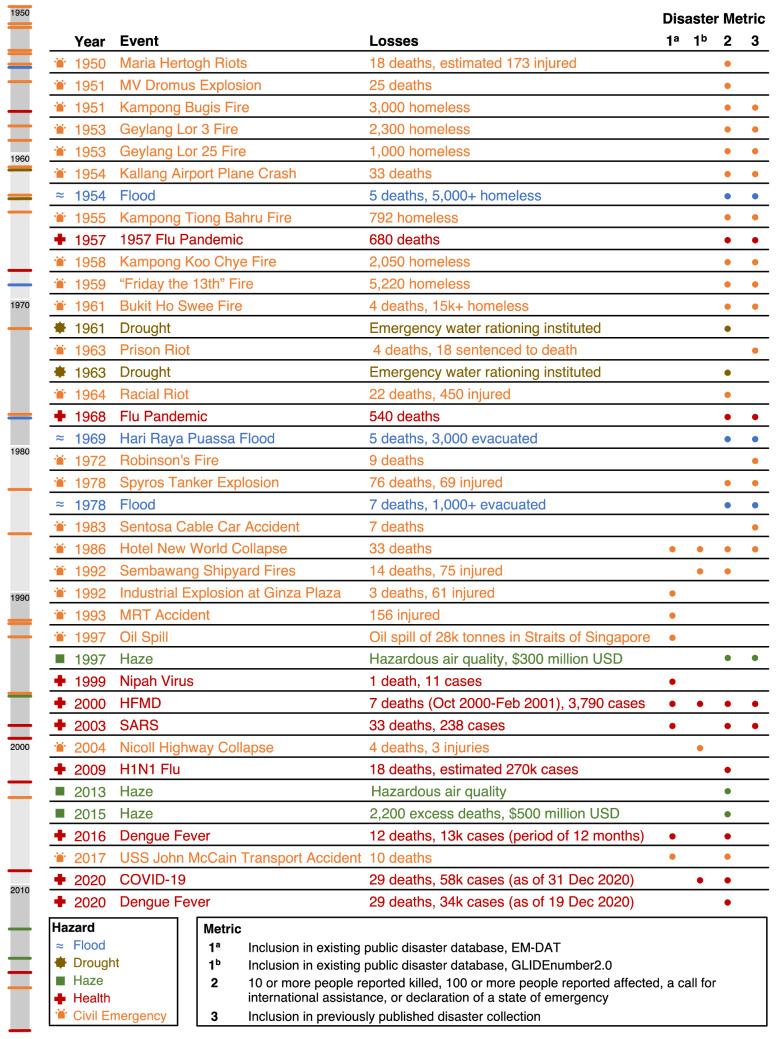
Past disasters of Singapore: Event type is indicated by the color and icon, and a timeline of events is included vertically to the left of the text. Qualifying disaster metrics are indicated by the dots for metrics 1a, 1b, 2, and 3. Note that none of the events prior to 1986 are included in either database. In total, these events represent an estimated 1628 deaths due to disasters (excluding excess deaths from the 2015 Haze event) in Singapore in this time period. References for each event are included in text

In total, we identified 39 events over 70 years (1950–2020) that met at least one of the metrics defined in Sect. 2. These events are sorted into five categories: flood, drought, haze, health, and civil emergency events. Of the 39 events, there are 3 flood events, 2 drought events, 3 haze events, 9 health events, and 22 civil emergency events. All events are included in Fig. 1, along with their respective originating database(s) or qualifying metric code(s). For events that meet multiple criteria within metric 2, the first qualifying metric is indicated in the event title (for example, if an event meets metrics 2a and 2c, we will attribute the metric to 2a).

### Floods

Singapore has had a number of flooding events in the past. Due to its natural topography and urban development, there are a number of low-lying, flood-prone areas that are also heavily populated and with considerable economic assets such as Orchard Road, Singapore’s primary high-end retail district, making impacts from floods particularly costly when they do occur (Friess and Oliver 2015). Floods typically have happened in the northeast monsoon period (November–December), and the effects of heavy rainfall can be exacerbated by coincidental high tides, which limit the capacity for channels to drain excess surface water (Friess and Oliver 2015). Three of the most disastrous floods in Singapore’s past include the floods of 1954, December 1969, and December 1978, all of which coincided with high tides (Friess and Oliver 2015). Though flooding continues in flood-prone regions such as Orchard Road (surrounded by hills and urbanized neighborhoods with limited green reserves for water absorption), more recent floods have not been as widespread and their impacts have mainly been due to economic damage from waterlogged assets and buildings.

Much of this improvement is the result of significant flood mitigation efforts in Singapore’s urban planning, including increased channel dimensions, additional water catchment capacity, and increased flood barrier and pump provisions (Friess and Oliver 2015). The effect of these mitigation measures is significant: the current flood-prone areas as identified by Singapore’s Public Utilities Board have been reduced from about 32 km^2^ (3200 ha) in the 1970s to 0.29 km^2^ (29 ha) in 2018 (PUB 2017, 2020a). These mitigation measures are currently being reviewed as a result of recent climate change-related scenario planning, with options such as empoldering and increased land reclamation also being considered (The Straits Times 2019).

#### Flood of 1954 (Meets Metrics 2b, 3)

Heavy rains from October to December of 1954 caused serious flooding in areas such as Bedok, Potong Pasir, Braddell Road, Long Tai Seng, and Geylang Serai in December 1954. The main four day storm flooded 25 km^2^, affecting 50,000 people and resulting in 5 fatalities (Friess and Oliver 2015). About 5000 people were rendered homeless as a result of these floods (The Straits Times 1954a). Additionally, rail travel was also closed during the height of the storm for 10 days in December (The Straits Times 1954b, 1954c, 1954d).

#### Flood of 1969 (Meets Metrics 2b, 3)

The floods of 1969 were described as the worst in 35 years (The Straits Times 1969). During this event on 10 December 1969 (on Hari Raya Puasa), 467 mm of rainfall fell in 17 h, and coincided with high tide; the result was 27 km^2^ flooded, roads submerged under 2 m of water heavily affecting safety and transport, the death of 5 people, and the evacuation of 3000 people (Friess and Oliver 2015). Helicopters were used to evacuate individuals trapped on their home roofs and stuck in trees (The Straits Times 1969).

#### Flood of 1978 (Meets Metrics 2b, 3)

Following heavy flooding in November of 1978, December struck again with severe floods with the wet northeast monsoon season, culminating in 512 mm of rain in a 24-h period (2–3 December 1978) coinciding with high tide (Friess and Oliver 2015). For perspective, the average rainfall in Singapore is about 2400 mm per year, meaning this single storm carried over 20% of a typical year’s rainfall. This flood disaster covered 31 km^2^, especially the Potong Pasir, Woodlands, Braddell Road, and Changi neighborhoods, claiming 7 lives and completely submerging homes (Friess and Oliver 2015; Lai and Tan 2015).

### Drought

Southeast Asia, including Singapore, is affected by the El Niño Southern Oscillation and the Indian Ocean Dipole, which are both large-scale atmospheric circulations that result in prolonged, warmer-than-normal drought conditions to the region (Tan et al. 2019). As an island-state with 5.7 million people and a total area of about 700 km^2^ (Singapore Land Authority 2019) with limited availability of natural water catchment, drought vulnerability and water management have been a primary concern for Singapore (Tortajada et al. 2013). Pre-independence in 1961 and 1962, Singapore negotiated favorable agreements for the transfer of water through three large pipelines across the 2 km causeway between Johor (Malaysia) and Singapore; the first agreement expired in 2011 and the second is valid until 2061 (Tortajada 2006). However, this agreement has been the source of political tension (Ewing and Domondon 2016). Early in postwar Singapore’s “water story” were two major drought events in 1961 and again in 1963 that both called for emergency measures for water conservation (Tan 2015).

#### Drought of 1961 (Meets Metric 2d)

Starting on 1 September 1961, Singapore instituted water rationing such that the water supply could be cut off at fixed times for 6 h at a time, 4 days per week, on a predetermined schedule (The Straits Times 1961b). After 4 months, the restriction was lifted on 26 January 1962 after reservoirs returned to full capacity following heavy rainfall, brought by the expected northeast monsoon season, in January 1962 (The Straits Times 1962).

#### Drought of 1963–1964 (Meets Metric 2d)

From 23 April 1963 through 28 February 1964, Singapore implemented emergency water rationing measures in response to drought conditions (The Straits Times 1963, 1964). During this period, water supply was cut off for residents up to 12 h at a time, and the government urged the public to conserve water. Water rationing ended after reservoir levels were once again sufficient, following sustained heavy rainfall in late February 1964 (The Straits Times 1964).

### Haze

“Haze”—the widely used term in Southeast Asia for fire-related regional, large-scale air pollution caused by clearing of forests and agricultural land—can pose serious health and economic risks (Emmanuel 2000). Smoke haze carries particulate matter that can increase acute health issues such as asthma, pulmonary infection, and eye and skin irritation, as well as other health impacts (Johnston et al. 2012; Lai and Tan 2015). The presence of haze particulates also limits visibility, which can result in restricted land, air, and marine transportation (Heil and Goldammer 2001). Overall, haze can impact the economy through healthcare costs, short-term tourism effects, travel disruptions, and production losses. Though Singapore has a longer history of haze dating back to the 1970s (Heil and Goldammer 2001), the country saw its first haze disaster in 1997, and this was followed again in 2013 and 2015. Haze is a transboundary issue between the countries of Southeast Asia, with fires originating in one country and particulates affecting neighboring countries, and can strain or even threaten diplomatic relations between countries (Forsyth 2014).

#### Haze of 1997 (Meets Metrics 2b, 3)

The haze in Singapore was a result of an estimated 45,600 km^2^ of vegetation burned in Indonesia in 1997 (Heil and Goldammer 2001). At the same time, an abnormal drought period due to the El Niño Southern Oscillation exacerbated the transport of haze to large areas within Southeast Asia, including Singapore (Koe et al. 2001). The effects were most widely felt in Singapore from the end of August to the beginning of November, as a result of prevailing northwest monsoon winds (Emmanuel 2000; Lai and Tan 2015). The air quality was in the “unhealthy” or “hazardous” range (pollutant standards index (PSI) > 100) 12 times during this period, reaching a maximum three hour average of 226 PSI on 18 September 1997 (Lo 1997). This event prompted the creation of a haze action plan by the Environment Ministry of Singapore (Nathan 1997). The economic cost of haze from this period is estimated to be over SGD 400 million (about USD 300 million) (Lee 2015).

#### Haze of 2013 (Meets Metric 2b)

In June 2013, wildfires spread across Indonesia’s island of Sumatra, this time decoupled from an El Nino year. This created one of the worst haze episodes in Singapore, with the 3-h moving average PSI hitting 401 (“hazardous” is 201–500; 500 is the index ceiling), and the 24-h moving average reported at 246 (Velasco and Rastan 2015). This event had milder economic impacts than the 1997 event, estimated at SGD 70 million (USD 50 million), though its unusual timing in June and extreme PSI peaks made this haze period particularly notable (The Straits Times 2015).

#### Haze of 2015 (Meets Metric 2a)

In September–October 2015, fires again burned in Indonesia, on the islands of Sumatra and Kalimantan. The effects of the 2015 haze episode were again exacerbated by drought conditions from El Niño (Koplitz et al. 2016). This episode is considered one of the most serious due to its prolonged duration from mid-September through the end of October. This time, the PSI lingered in “hazardous” conditions (24-h 322 PSI) long enough to warrant the first-time haze-induced closure of all primary and secondary schools on 25 September (Lee 2015). This episode was estimated to cost Singapore about SGD 700 million (about USD 500 million) (Chin 2016), and is responsible for the estimated excess deaths of 2200 (600–3800 95% confidence interval) Singaporeans in the following year (Koplitz et al. 2016).

### Health Disasters

As one of the densest countries in the world (United Nations 2019) and as a tropical island nation, Singapore is especially vulnerable to infectious health disasters. This is reflected in the number of fatalities originating from health disasters (up to 680 lives lost in a single epidemic) as compared to any other disaster category in the last 70 years, where the maximum lives lost in a single event was 76 lives (see Sect. 3.5.8 Spyros Disaster). The impactful epidemic episodes are included here.

#### 1957 Influenza (Meets Metric 2a, 3)

Historically, a number of influenza epidemics have inflicted Singaporeans, but in April–May 1957, Singapore saw the biggest outbreak of influenza since 1918 (Lee et al. 2008). Official statements at the time reported that in May 1957, there were 77,211 cases and 28 deaths (Ministry of Health, Singapore 1957). However, Lee et al. (2008) suggest from an excess mortality calculation that this number could be as high as 680 deaths for the same period, and official reported numbers have since been updated to 680 when referencing this past event (Ministry of Home Affairs, Singapore 2009).

#### 1968 Influenza (Meets Metric 2a, 3)

The 1968 influenza outbreak was reported to have spread from Hong Kong in early August. The outbreak lasted a few weeks, peaking between 16–25 August 1968. The excess mortality is reported to have been around 540 deaths in that period (Lee et al. 2008; Ministry of Home Affairs, Singapore 2009). The pandemic was the result of an antigenic shift in the influenza A virus (Lee et al. 2007).

#### Nipah Virus in 1999 (Meets Metric 1a)

Thought at first to be Japanese Encephalitis, the now-identified Nipah virus spread from Malaysia and infected 11 Singaporean abattoir workers, with one fatality. The illness spread from commercially raised pigs, and only those who had recently been exposed to pigs fell ill. There were no documented cases of human-to-human transmission (MMWR 1999).

#### Hand, Foot, and Mouth Disease in 2000 (Meets Metrics 1a, 1b, 2b, 3)

The largest outbreak of hand-foot-mouth disease (HFMD) took place in September to December 2000. This outbreak infected 3790 young children (children under 5 years old are most susceptible) and resulted in four deaths (Ang et al. 2009). In October 2000, HFMD was designated as a legally notifiable disease, meaning it is required by law for the disease to be reported to government authorities so that it can be effectively monitored for possible outbreaks (Singapore Government Media Release 2000). Three additional deaths followed in January and February 2001 (Ang et al. 2009).

#### SARS in 2003 (Meets Metrics 1a, 2a, 3)

The first case of the severe acute respiratory syndrome (SARS) originated from a Singaporean returning from Hong Kong in March 2003 (Lai and Tan 2015). By the end of July 2003, Singapore reported 238 probable SARS cases and 33 deaths (The Straits Times 2013). During the course of the outbreak, the Singapore government implemented a number of public policies, including social distancing, quarantine with closed circuit camera monitoring to ensure compliance, and isolation to mitigate transmission risk (Lai and Tan 2015). Alongside fatalities and cases, Singapore’s economy was heavily impacted by work disruptions from employees and by a sharp decline in the demand side, particularly to the tourism, retail, hospitality, and transportation-related industries (Lai and Tan 2015).

#### H1N1 Flu Epidemic in 2009 (Meets Metric 2a)

The first H1N1 outbreak in Singapore was reported to have been detected in May 2009 (Cutter et al. 2010). Cutter also estimated that at least 270,000 persons in Singapore were affected between May and September 2009. There were three waves of cases, the first from May to June, mainly imported from the United States, the second in June from Australia, and the last wave from the end of June onwards, mainly imported from Southeast Asia. Morbidity and mortality during the H1N1 flu epidemic were generally low, with a total of 18 deaths reported in Singapore in 2009 (Cutter et al. 2010).

#### Dengue in 2016 (Meets Metrics 1a, 2a)

In February 2016, authorities forecasted a potentially historic high of 30,000 dengue cases (Tang 2016). The initial scare was due to a peak of 600 reported cases of dengue in January, historically a low period for dengue (Rajarethinam et al. 2018). However, in August, Singapore was faced with another mosquito borne illness, Zika, which was much less familiar to residents. The additional scare may have contributed to curbing the 2016 dengue outbreak by motivating residents to take mosquito mitigation efforts more seriously (Khalik 2016). The year ended with 13,085 dengue cases and 12 fatalities (Rajarethinam et al. 2018).

#### COVID-19 in 2020 (Meets Metric 1b, 2a)

In early 2020, Singaporean authorities were aware of the novel coronavirus and its associated disease, COVID-19, and implemented travel restrictions and contact tracing measures to contain the spread of the virus in its population after early cases were detected (Lee et al. 2020). While the country’s initial efforts to suppress the global pandemic through contact tracing and quarantines were promising, cases began to spike in late March 2020 as several clusters were identified in local places of worship and later in migrant worker dormitories. A nationwide lockdown was imposed as cases spiked to 40,000 within three months, and 29 fatalities had been recorded by 31 December 2020. However, due to a strict policy of containment, the vast majority of the 57,000 cases have been concentrated within Singapore’s migrant worker dormitories (over 95% of reported cases as of 11 September 2020) while “community” cases—those among Singapore residents not isolated in dormitories—have remained low enough for a phased reopening (Ministry of Health, Singapore 2020a, 2020b).

#### Dengue in 2020 (Meets Metric 2a)

As of 18 December 2020, there have been over 34,800 reported cases of dengue in Singapore and 29 deaths in 2020 (Qing 2020). The number of weekly cases has set a new record for the number of cases per week in the 30th week of 2020 with 1792 reported cases (National Environment Agency 2020b). As this outbreak’s dominant strain, dengue virus serotype 3, has not been the dominant strain in Singapore for over three decades, there is low community immunity (National Environment Agency 2020a). A concurrent COVID-19 crisis impacted response and public awareness of the outbreak (Lim 2020). Additionally, the main vector of dengue primarily bites indoors during the day; the number of dengue cases was likely exacerbated by the stay-at-home measures in place during the most restrictive phase of the COVID-19 Circuit Breaker (April 7—June 1 2020) (Khalik 2020).

### Civil Emergencies

**Fig. 2:**
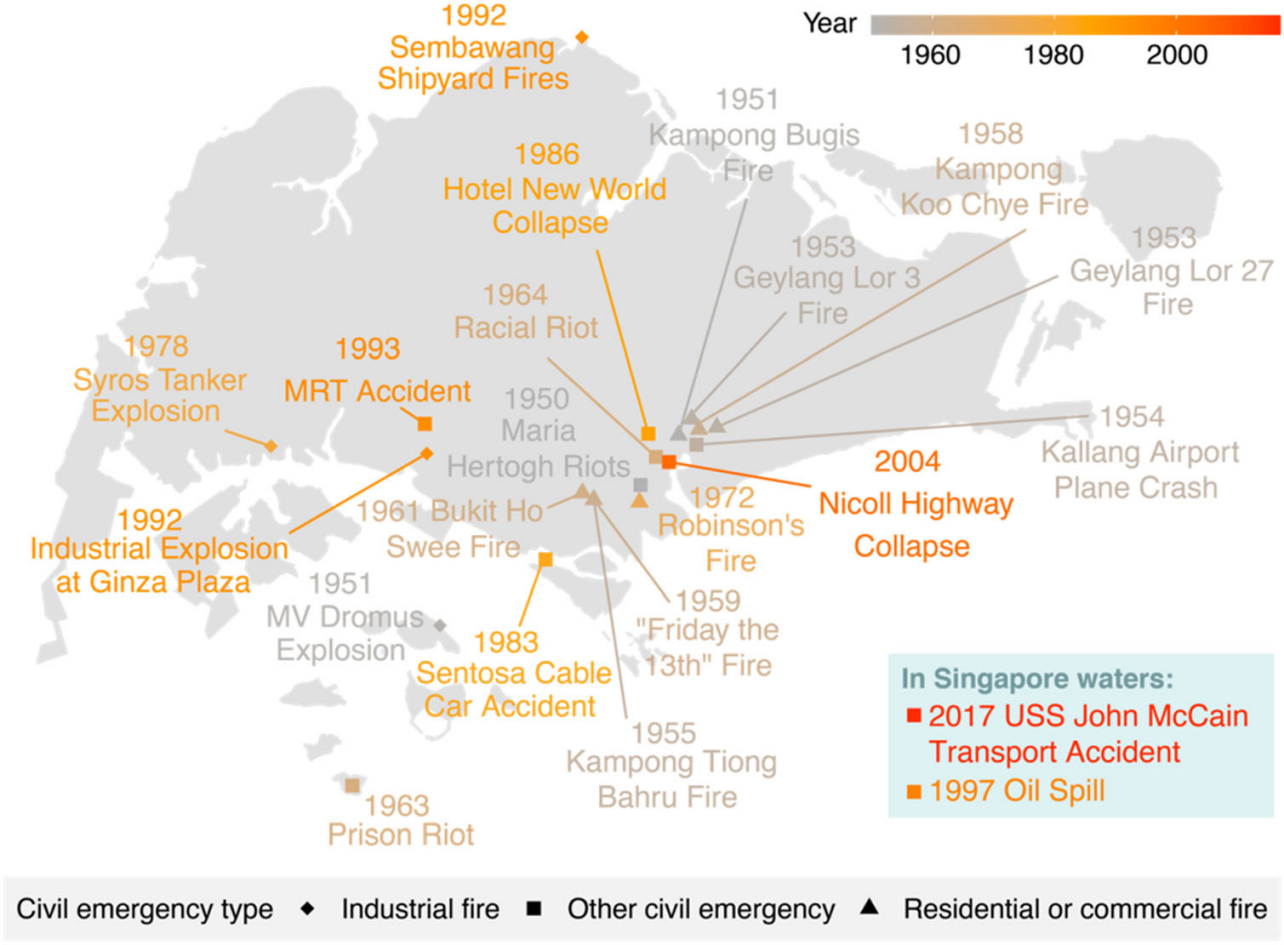
Map of Singapore’s past civil emergencies. Civil emergencies are categorized into three subtypes: industrial fire, residential or commercial fire, and all other civil emergencies. Time is indicated in the label of each event and by a gradient color scale from grey to red, where red events are more recent

Civil emergencies are defined as any sudden incident involving (1) large-scale loss of life or damage to property; (2) a major incident with the potential to escalate in scale; (3) major national, diplomatic, or political implications; or (4) requiring multi-agency response to manage the events arising from the incident. This can include fires, explosions, oil spills, or hazardous materials incidents (Government of Singapore 1986; Yung 2012). They may also include floods, storms, or other impacts from natural hazards, but are not included in this section because they have been discussed above. Of the five categories (flood, drought, haze, health, and civil emergency), in Singapore, this category has the highest number of events. These are generally considered to be anthropogenic disasters or mass emergencies. The past civil emergency disasters from 1950 to 2020 are shown in Fig. 2.

Among the civil emergencies that have affected Singapore, fires—especially in urban settlements—have played an outsized role in shaping the city-state’s approach to housing, emergency services, and public space. Historian Loh Kah Seng draws from survivors’ accounts, oral histories, and archival research to highlight how residents of urban *kampongs* (small settlements and towns) in the 1950s and 1960s lived with constant and frequent fire hazards, which catalyzed much of post-independence Singapore’s transition from “squatters to citizens” through massive public housing investment and relocation of households rendered homeless by such fires (Loh 2009, 2013). These emergencies affected thousands of people, with the Bukit Ho Swee fire of 1961 being the most commonly remembered as it rendered nearly 16,000 people homeless (Loh 2013). Most significantly for our discussion, these fires are omitted from major databases despite affecting thousands of people and leading to significant action in risk reduction measures and systemic reforms.

#### Maria Hertogh Riots in 1950 (Meets Metric 2a)

Between 11–13 December 1950, Singapore experienced an “outbreak of mass violence” after the result of a protracted and controversial legal battle between the birth parents and adoptive family of Maria Hertogh (also known as Nadra binte Ma’arof) (Aljunied 2009). On the 11th, demonstrators opposed to the Supreme Court ruling that custody of Maria be given to her birth parents gathered around the Supreme Court by St. Andrew’s Road. The demonstration escalated into a riot after several incidents, including the assault of a volunteer police officer. Violence escalated over the subsequent hours and days, with machine guns deployed and police officers opening fire as curfews were declared. By noon on the 13 December, 18 people were dead with an estimated 173 injured, along with 1168 arrested for looting and 500 detained under Emergency Regulations (National Library Board Singapore 2014).

#### MV Dromus Explosions in 1951 (Meets Metric 2a)

In the night of 19 August 1951, MV Dromus, a 1930s British oil tanker owned by Anglo-Saxon Petroleum, exploded and caught fire just after midnight on the island of Palau Bukom (The Straits Times 1951b). Shortly thereafter, a second explosion worsened the fire. Ultimately these explosions claimed 25 lives, but there was confusion and inconsistency in the number of missing persons, and reported deaths for many days following this disaster (The Straits Times 1951a).

#### Airplane Crash at Kallang Airport in 1954 (Meets Metrics 2a, 3)

On 13 March 1954, Kallang Airport had its first major air disaster when a plane missed the runway and crashed, resulting in 33 fatalities (The Age 1954; The Straits Times 1984, 1986). The passengers were trapped inside the flaming cabin, which caught on fire from the crash, with the crew and firefighters unable to cut away the cabin in time. Following this disaster, significant improvements to firefighting equipment and training were implemented to avoid a repeat of these circumstances (The Straits Times 1956).

#### Kampong Fires in the 1950s and the Bukit Ho Swee Fire in 1961 (Meets Metrics 2b, 3)

On 25 May 1961, which happened to be Hari Raya Haji that year, the biggest fire in Singapore history broke out in the largely squatter settlement Bukit Ho Swee area (The Straits Times 1961a). There were 4 casualties, and over 0.4 km^2^ was destroyed, leaving nearly 16,000 people homeless (The Straits Times 1984, 1986; HistorySG 2014).

Crucially, the Kampong Bukit Ho Swee fire was not an isolated incident but rather one of the last of a string of fires since the Kampong Bugis fire in 1951. Just two years prior in 1959, the infamous “Friday the 13th” fire in Kampong Tiong Bahru displaced 5220 people, while in 1958 the Kampong Koo Chye fire led to the first formation of volunteer firefighting units in urban settlements. Historians of Singapore have combined archival and oral histories research highlighting the broad spatial spread of fires, tracing postwar Singapore from the 1951 Kampong Bugis fire to the 1961 Bukit Ho Swee fire (Loh 2009). These residential fire events are summarized in Table 2.

**Table 2:** Summary of the Kampong fires throughout the 1950s, culminating in the 1961 Bukit Ho Swee fire that prompted massive public housing investment

Year	Place	Affected persons
1951	Kampong Bugis	3000 homeless
1953	Geylang Lorong 3	2385 homeless
1953	Geylang Lorong 25	1000 homeless
1955	Kampong Tiong Bahru	792 homeless
1958	Kampong Koo Chye	2050 homeless
1959	“Friday the 13th” fire in Kampong Tiong Bahru	5220 homeless
1961	Kampong Bukit Ho Swee	15,694 homeless; 4 dead

*Source* Events are compiled from Loh (2009)

#### Prison Riot of 1963 (Meets Metric 3)

On 12 July 1963, 70 or more inmates of the experimental no-bars prison island on Pulau Senang rioted (The Straits Times 1984, 1986; Li 2015). Four were killed, including the penal colony superintendent. A 64 day trial of 59 inmates ensued, and ultimately 18 were sentenced to death (The Straits Times 1984). The penal colony was not reopened.

#### Racial Riots in 1964 (Meets Metric 2a)

The July 1964 Sino-Malay riots are considered one of the worst in Singapore’s history. According to official statistics, 22 people were killed and another 454 injured. Interestingly, they are neither mentioned in the disaster collection put together by *The Straits Times* in 1984, nor included in any of the databases or other collections, though they are a prominent part of Singapore’s history and collective memory (Cheng 2001). There was another resurgence of riots later in September 1964.

#### Robinson’s Fire in 1972 (Meets Metric 3)

On 21 November 1972, a fire destroyed a Robinson’s department store in Raffles Place. Nine people perished; of the nine, eight were trapped in the elevator (The Straits Times 1984, 1986). The cause of the fire was discovered to be a short circuit due to overloading in the four-story building (The Straits Times 1984).

#### Spyros Disaster in 1978 (Meets Metrics 2a, 3)

On 12 October 1978, 76 of 167 people working on the 35,700-ton Greek tanker Spyros died due to a tanker explosion at Jurong Shipyard (The Straits Times 1984, 1986; Lai and Tan 2015). Causes of death included severe burns, carbon monoxide poisoning, suffocation, drowning, and burns-related complications. An additional 69 people were injured.

#### Sentosa Cable Car in 1983 (Meets Metric 3)

The derrick of an oil drilling vessel struck the Sentosa cable car cableway on 29 January 1983, causing two cable cars to drop 55 m into the sea (The Straits Times 1984, 1986). There were seven deaths, and 14 people rescued, including one two-year old boy, from the sea (The Straits Times 1986).

#### Hotel New World Collapse in 1986 (Meets Metrics 1a, 1b, 2a, 3)

On 15 March 1986 at 11:25 a.m., the six-story Hotel New World (Lian Lak Building) suddenly collapsed (The Straits Times 1984, 1986; Lai and Tan 2015). A total of 33 people died and 17 were rescued. The resulting inquiry found that the building had been designed by unqualified persons, and that an excess of 100-ton of live load had been added to the building after construction (Thean et al. 1987). There were several signs of structural failure leading up to the collapse, including signs of new formed diagonal cracks and sounds of cracking from columns; however, the building was not evacuated. This is the worst building collapse in Singapore’s history, and rescue forces were underprepared to respond in a disaster of this scale. Following this collapse, several changes were made to Singapore’s building design and construction regulations (Thean et al. 1987).

#### Industrial Fires at Sembawang Shipyard in 1992 (Meets Metrics 1b, 2a)

On Sunday, 12 July 1992 at about 11:00 a.m., a fire broke out aboard MT *Stolt Spur* at Sembawang Shipyard (Magnus 1992). Six workers died and 61 others were injured. Just a few months later on 27 November 1992 at about 2:20 p.m., a fire on board the *Indiana*, a chemical tanker, broke out (Magnus 1993). Eight workers died, and 14 others injured. These incidents helped push for better workplace safety and health regulations in Singapore’s industrial sector, such as the Factories (Crane Drivers and Operators) Regulations of 1993, and the creation of an online computerized system for inspection records of pressure vessels in 1992 (Singapore and Occupational Safety and Health Division 2016).

#### Industrial Explosion at Ginza in 1992 (Meets Metric 1a)

An explosion and injuries were reported at 2:13 p.m. on 7 August at Ginza Plaza Podium, a three story shopping block with one basement level. When responders arrived at the scene at 2:18 p.m., a gas pipe in the ceiling of the basement was found on fire. Three dead bodies were found pinned under concrete slabs, and there were an additional 61 injured (SCDF 2018a).

#### MRT Accident in 1993 (Meets Metric 1a)

The first accident on the mass rapid transit (MRT) system in Singapore occurred on 5 August 1993. The MRT started operation in 1987. There were 156 injuries from the front-to-back collision between two trains at the Clementi Station. The collision was caused by a 50-L oil spill from a maintenance locomotive, which had a broken rubber gasket that caused oil to spill on the track earlier that morning, and was not cleaned in time to prevent this accident (Yuen 2004).

#### Oil Spill in 1997 (Meets Metric 1a)

On 15 October 1997, two tankers (the 129,702 gross ton Thai *Orapin Global* and 75,426 gross ton Cyprus *Evoikos*) collided and spilled 28,463 tons of oil in the Singapore Strait. The Singapore government sought emergency aid from the Japanese government. This was an environmental disaster for mangrove forests and coral reefs near Raffles Lighthouse (MOFA 1997), and is considered one of the worst spills in the history of Singapore. As an example of a coinciding event, cleanup efforts were adversely affected by the simultaneous haze disaster (Siang 2000).

#### Nicoll Highway Collapse in 2004 (Meets Metric 1b)

Part of the Nicoll Highway collapsed on 20 April 2004 over the MRT Circle Line Tunnel construction, which was ongoing underneath it at the time, resulting in a four day rescue operation, three injuries, and four fatalities (SCDF 2018b). The highway was closed for seven months before it was reopened to road users. The damage costs from this disaster are estimated to be in the millions, and the collapse was concluded to be due to “critical design errors” resulting in the failure of the earth-retaining wall system (Ministry of Transport, Singapore 2005).

#### USS McCain Transport Accident in 2017 (Meets Metrics 1a, 2a)

On 21 August 2017, USS John S McCain turned suddenly and entered the path of oil tanker *Alnic MC* in the Singapore Strait (Kaur 2018). A collision shortly followed and resulted in the deaths of 10 American sailors. An inquiry resolved that this was mainly the responsibility of the commanding officer of USS John S McCain for exercising poor judgement and decision making (Kaur 2018).

## Discussion

We have collected this set of 39 events in order to understand the past disasters of Singapore and the completeness and state of its representation in disaster databases. Prompting the creation of this work was the realization that none of the previous disaster studies, databases, or coverage of Singapore has been sufficiently representative of the extent of events that have occurred in Singapore. We identify four lessons from cataloging the past disasters of postwar Singapore: (1) the quantitative extent of the data gap on Singapore’s disasters; (2) past disasters as drivers of policy and mitigating action; (3) consistency in vulnerabilities magnified by past disasters; and (4) limitations of a disaster catalog for future risk.

### Quantifying the Disaster Data Gap in Singapore

**Fig. 3:**
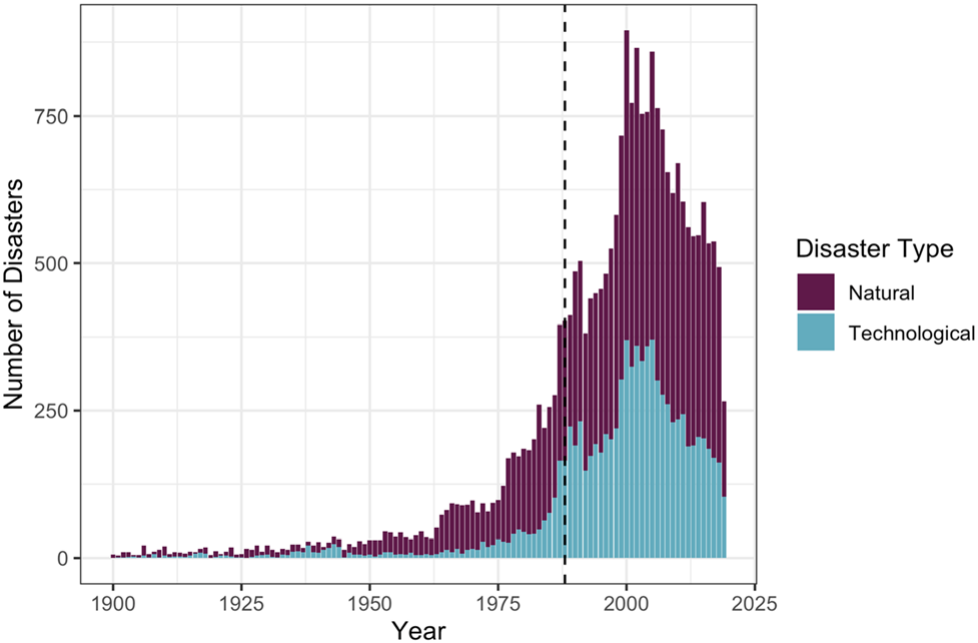
All events in EM-DAT database, by year (CRED n.d.). The database covers 1900 to present day (these records were accessed on 4 October 2019). The database was established in 1988 (denoted by the vertical black dashed line) at which time we see a substantial increase in records present in the database. This is consistent with the EM-DAT coverage of Singapore, whose first record appears in 1986, even though events prior to this date would qualify to be included in the database

Twelve events were included because of their existing records on international databases, EM-DAT and GLIDEnumber. EM-DAT has nine records, while GLIDEnumber has five records, but only two of those events appear in both; this suggests that, though GLIDEnumber is meant to be consistent with EM-DAT, in practice this has not been achieved for Singapore. As shown in Fig. 1, EM-DAT, whose coverage is meant to begin in 1900, does not include any records for Singapore events prior to 1986, even though events such as the floods of 1954, 1969, and 1978 all meet multiple disaster criteria as set forth by the EM-DAT definition. These missing events point to a larger trend within the EM-DAT database: as shown in Fig. 3, the number of records contemporary with the establishment of the database in 1988 to present day greatly outnumber earlier records, in particular, prior to 1960. Rather than suggesting a dramatic increase in disasters in the last few decades, this suggests that EM-DAT’s scarcity of records for Singapore pre-1986 is reflected across the database as well. Other scholars have also identified EM-DAT’s bias for events that date past 1995, seven years after its establishment (Panwar and Sen 2019).

Adhering to a global definition of disaster may also not reflect the risk tolerance of a specific country. To address this, the DesInventar methodology for disaster information management specifically emphasizes accounting for small to medium disasters in order to enable risk analysis at the community level (UNDRR 2019). However, as mentioned, available DesInventar Sendai records do not have any events for Singapore (UNDRR 2015a).

Another 18 events were included under the third metric, having already been identified in other disaster catalogs for Singapore. Of the published disaster collections, they tend to skew closer to their own publication date. Hence, *The Straits Times* collection includes events mostly in the decade preceding its publication (initially 1984, then run again in 1986), and the study by Lai and Tan in 2015 is largely concentrated in the 1990’s and 2000’s. The hazard specific collections tended to have better coverage on their single hazard (Friess and Oliver 2015 for floods, Lee et al. 2008 for influenza epidemics before 2009). Thus, this serves to consolidate these disaster histories into a single collection for modern Singapore.

Finally, nine events had not been included in any disaster collections that we encountered, though they met at least one of the EM-DAT definitions outlined in Table 1. These include the Maria Hertogh riots in 1950, the MV Dromus explosion in 1951, the fires that led up to the Bukit Ho Swee fire of 1963, and the racial riot of 1964. Riots are not a part of the EM-DAT scope, though they could have been within the scope of other Singapore collections reviewed.

### Past Disasters as Drivers of Policy and Mitigating Action

More broadly, this process of developing a more comprehensive catalog serves as an important counterpoint to the narrative that disasters are “new” or “unprecedented.” While it is indeed true that the drivers of natural hazard-related disasters are becoming more urgent and creating even more vulnerability (UNDRR 2015b), one important insight provided by implementing this search process in Singapore is that the past is not as empty or disaster-free as initially imagined. Rather, the fact that Singapore’s catalog expanded so thoroughly after our search highlights how disasters were actually more frequent in the past than often cataloged, and a process of generational and institutional learning has since allowed for the incorporation of these events into other parts of the historical record, risk management practice, and public policy.

Consider the drought hazard, for example. Though Singapore is positioned to be vulnerable to drought, its “water story” is today seen as one of success (Jacobson 2012; Tortajada et al. 2013). Driven by the desire for eventual water independence, Singapore has a system of four main sources of water, referred to as the “four national taps”: water catchment, which has increased from half of Singapore’s land area (2011) to two-thirds of the land area (2019) and a total of 17 water reservoirs; imported water, which is guaranteed until 2061; NEWater, which recycles reclaimed water; and desalinated water (PUB 2020b). Singapore has also employed strategies to manage water demand and has seen a decline in water use per capita from 165 L per person in 2000 to 141 L per person in 2018 (PUB 2020b). One major strategy for water conservation has been through public involvement through education and information and awareness campaigns (Tortajada and Joshi 2013). Though there have been drought events since the 1961 and 1963 events (for example, 1971, 1990, 2014, 2015–2016), one indicator of success from Singapore’s water strategy is that the country has not had to call for water rationing measures to manage the impacts of drought conditions since the 1960s (The Straits Times 1971; Chang and Irvine 2014; Tan 2015; Chuah et al. 2018).

There have also been a number of industrial disasters that have resulted in collapses, fires, deaths, and injuries. In some cases, these disasters have translated directly into action, often new regulations enforcing training and other preparedness measures by the Singapore government such as the addition of firefighting equipment and training following the 1954 plane crash, modification of the Singapore building design and construction regulations following the 1986 Hotel New World collapse, and the adoption of workplace safety and health regulations such as the Crane Drivers and Operators Regulations of 1993. Some regulations have been enacted in law but are challenging to enforce, such as Singapore’s Transboundary Haze Pollution Act (Tan 2019).

In some cases, disasters omitted in databases have actually been crucial to formulating Singapore’s current risk landscape and are widely understood in local or cultural memory. For example, although Singapore’s EM-DAT record does not include the Maria Hertogh riots due to the nature of the emergency (political- or social-driven events are excluded) or the 1961 Bukit Ho Swee fire due to a record omission, both of these events have gone on to become critical parts of Singapore’s public policy casebook and have informed current approaches to risk management, and are even taught to children in schools. The Maria Hertogh riots, for example, are frequently invoked as a motivator of Singapore’s approach to managing community racial relations, promoting racial harmony, and de-escalating potential racial tensions (Aljunied 2009). Similarly, the Bukit Ho Swee fire has been incorporated into many narratives of nation-building and the role of public housing in public safety (Loh 2013).

Expanding the disciplinary toolbox in searching for previous disaster events allows researchers and practitioners of risk management to highlight the generational learning and risk mitigation practice that has developed using locally relevant categories that still broadly meet the intentions of international disaster catalogs and databases. The fact that this work to expand Singapore’s catalog yielded so many events to consider beyond those that are currently represented is particularly noteworthy, and suggests that similar searches in other contexts could yield significant results as well.

### Magnifying Existing Vulnerabilities

A more comprehensive catalog of Singapore’s past disasters also allows for patterns to be identified that would otherwise be challenging to notice with few events. One important finding from our search was that disaster fatalities and impacts were especially concentrated in specific communities such as migrant workers, the elderly, and the economically disadvantaged. This accords with findings from other contexts that highlight the existence of “vulnerability bearers” (Peterson 2019; Peek 2019) or emphasize the uneven distribution of disaster risk within populations (Wisner et al. 2004).

A few examples illustrate this. First, we highlight the parallels between the COVID-19 pandemic and the 1918 flu pandemic. Though the 1918 event is beyond the scope of the present work, the two events have many parallels considering their near 100 year difference in time: in both cases, migrant workers living in housing conditions of a poorer standard than the rest of the population suffered the most impacts including fatalities, stigmatization as disease carriers, and lost livelihoods (Ho 2020). Crucially, the state of these housing conditions is a reflection of social decisions and policies. Far from disease transmission being a result of “cultural factors” or poor individual hygiene, in both cases the underlying conditions governing migrants’ standard of living—investment in quality housing, for example—determined their exposure to these pathogens (Ho 2020). Similarly, the Bukit Ho Swee fire has been described as a watershed moment for Singapore’s urban housing landscape because of how the impetus of housing fire-displaced residents led to the first significant projects by the Housing Development Board (Loh 2013). However, accounts of victims also highlight how the communities exposed to fire were among the more economically disadvantaged, many of whom were pushed by economic hardship into more cramped living conditions without adequate fire prevention (Loh 2013).

While ours is a backwards-looking catalog of past events, contemporary risk analysis in Singapore highlights that some prior risks—such as vector-borne diseases like dengue, weather-related conditions like pollution, or illnesses like heat stroke—may be exacerbated in the future by climate change (Campbell-Lendrum et al. 2015). These vulnerabilities may also track onto economic and demographic disparities in working conditions, access to resources, and/or amenities like air-conditioning (Kjellstrom et al. 2017). More comprehensive catalogs therefore allow researchers and planners to see both change and continuity in future risk assessments.

### Limitations of a Disaster Catalog

A catalog of past events, such as this one, has limitations and invites critique, expansion, and broad engagement. First, this collection does not capture the range of impacts that could have transpired in any of these events. However, this collection can be used as a basis to explore slight changes to past events, and begin to understand the possible range of consequences using methods in counterfactual risk analysis (Lin et al. 2020). For example, the 2016 dengue outbreak may have been mitigated by the impending threat of Zika virus, moving residents of Singapore who are familiar with the threat of dengue but unfamiliar with the new dangers of Zika into action against mosquito breeding grounds. Second, a catalog also does not capture near-miss events—events that could breach one of the disaster metrics (such as the thresholds chosen here for metrics 1, 2, and 3), but just barely did not clear the threshold either because of spatial, temporal, or cultural factors. Finally, the catalog inherently cannot capture emergent or changing hazards (for example, climate change); additional tools (for example, Shepherd et al. 2018; Woo 2019; Lin et al. 2020) are needed for those kinds of horizon scanning and catalog expansion. However, this catalog is still a helpful tool to frame conversations around evolving hazards because the dynamics of vulnerability described in Sect. 4.3 may also be at play when considering future risk.

## Conclusion

For a country with limited number of disasters, this process of cataloging offers those in disaster science the rare opportunity to have a more complete record of past disasters, at least in the past generation (for example, we have chosen 1950). Through a detailed analysis of Singapore’s past disaster history from 1950 to the present day, we offer a quantitative data point to inform the number of events that may be missing from available disaster databases at the country scale and lessons learned from cataloging these events.

In total, we identified 39 past disasters (three flood events, two drought events, three haze events, nine health events, and 22 civil emergencies) that had been included in a publicly available disaster database (metric 1), met at least one of the quantitative EM-DAT disaster definitions (metric 2), or had been identified by other researchers as a disaster in previously published archives and collections on disasters in Singapore (metric 3). The EM-DAT database only contained nine records of past disasters within the same period, with the next most complete collection having eight records (that is, Lai and Tan 2015). This catalog of modern Singapore’s disasters fills a gap in the literature and provides a basis from which to examine Singapore’s past, current, and future risk reduction and preparedness policies and activity.

A complete disaster catalog of past events is integral to meeting the international goals of the SFDRR and is a necessary component for justifying investments in mitigation and building a more resilient tomorrow. Past events—in particular, past disasters—leave their legacies on current policies, risk management practices, and emergency preparedness, and affect our current and future perceptions of risk. This work provides a key data point for disaster risk researchers, governments, nonprofit organizations, insurance industry, community organizations, and other stakeholders to better understand the number of potentially “invisible” but influential events that exist for Singapore and may exist for other countries globally.
